# Can community health officer-midwives effectively integrate skilled birth attendance in the community-based health planning and services program in rural Ghana?

**DOI:** 10.1186/1742-4755-11-90

**Published:** 2014-12-17

**Authors:** Evelyn Sakeah, Lois McCloskey, Judith Bernstein, Kojo Yeboah-Antwi, Samuel Mills, Henry V Doctor

**Affiliations:** Social Science Unit, Navrongo Health Research Centre, Upper East Region, Navrongo, Ghana; Department of Community Sciences, Boston University School of Public Health, Boston, MA USA; Department of Global Health, Boston University School of Public Health, Boston, MA USA; Health, Nutrition, and Population, Human Development Network, The World Bank, Washington, DC USA; Integrated Programme and Oversight Branch, Division for Operations, United Nations Office on Drugs and Crime, Abuja, Nigeria

**Keywords:** Community-based service delivery, Ghana, Maternal mortality, Skilled birth attendance

## Abstract

**Background:**

The burden of maternal mortality in sub-Saharan Africa is very high. In Ghana maternal mortality ratio was 380 deaths per 100,000 live births in 2013. Skilled birth attendance has been shown to reduce maternal mortality and morbidity, yet in 2010 only 68 percent of mothers in Ghana gave birth with the assistance of skilled birth attendants. In 2005, the Ghana Health Service piloted a strategy that involved using the integrated Community-based Health Planning and Services (CHPS) program and training Community Health Officers (CHOs) as midwives to address the gap in skilled attendance in rural Upper East Region (UER). The study assesses the feasibility of and extent to which the skilled delivery program has been implemented as an integrated component of the existing CHPS, and documents the benefits and challenges of the integrated program.

**Methods:**

We employed an intrinsic case study design with a qualitative methodology. We conducted 41 in-depth interviews with health professionals and community stakeholders. We used a purposive sampling technique to identify and interview our respondents.

**Results:**

The CHO-midwives provide integrated services that include skilled delivery in CHPS zones. The midwives collaborate with District Assemblies, Non-Governmental Organizations (NGOs) and communities to offer skilled delivery services in rural communities. They refer pregnant women with complications to district hospitals and health centers for care, and there has been observed improvement in the referral system. Stakeholders reported community members’ access to skilled attendants at birth, health education, antenatal attendance and postnatal care in rural communities. The CHO-midwives are provided with financial and non-financial incentives to motivate them for optimal work performance. The primary challenges that remain include inadequate numbers of CHO-midwives, insufficient transportation, and infrastructure weaknesses.

**Conclusions:**

Our study demonstrates that CHOs can successfully be trained as midwives and deployed to provide skilled delivery services at the doorsteps of rural households. The integration of the skilled delivery program with the CHPS program appears to be an effective model for improving access to skilled birth attendance in rural communities of the UER of Ghana.

## Background

High maternal mortality is a grave concern worldwide. The global burden of maternal death is enormous, especially in less developed countries[1]. According to WHO, UNICEF, UNFPA, and The World Bank, the number of maternal deaths globally in 2013 was 289,000—a decrease of 45 percent from the 1990 levels (523,000)[2]. However, 56 percent of global maternal deaths occur in sub-Saharan Africa. Ghana is among the countries in sub-Saharan Africa with the highest overall maternal mortality ratios (MMR) at 380 deaths per 100,000 live births in 2013[2].

Multi-pronged strategies are needed to lower MMRs in countries such as Ghana. Among them, improved family planning, safe abortion or adequate post-abortion care, improved coverage and quality of skilled attendance at birth, and access to emergency obstetric care to address maternal mortality[3, 4]. Experts agree that access to skilled attendants at birth is a key component for nations committed to preventing maternal deaths; indeed, the proportion of births with skilled attendants is one of the key indicators for monitoring progress towards the achievement of Millennium Development Goal (MDG) 5[3, 4].

However, progress in achieving MDG 5 is still slow in sub-Saharan Africa, as is the move toward universal skilled birth attendance[5]. In Ghana overall, half of childbearing women give birth with a skilled attendant; and the rural–urban gap is wide (88% in urban areas and 54% in rural areas)[6].

The term "*skilled attendant*" is defined as an accredited health professional—such as a midwife, doctor or nurse—who has been educated and trained to proficiency in the skills needed to manage normal (uncomplicated) pregnancies, childbirth and the immediate postnatal period, and to identify, manage and refer complications in women and newborns[7]. Unfortunately, skilled delivery programs suffer from serious shortages of nurses, as well as professional midwives in Africa[8]. The ratio of nursing and midwifery staff in Africa is only 11 per 10,000 populations, compared with 79 per 10,000 in Europe[1]. In Ghana; attrition of health personnel to more developed countries continues to diminish the supply of providers, including nurses and midwives, needed to meet the population demands in the country[8]. In view of these constraints, national health systems have adopted strategies of a "task delegation", "task shifting" or "task sharing", a practical response to the skill shortages in the developing world[8, 9]. Evidence has shown that lower level cadres of healthcare staff can substitute for highly trained health professionals, such as doctors or certified nurse midwives for the performance of specific tasks that are in high demand[11–13]. For instance, in Mozambique medical assistants have been trained to perform surgical procedures in rural areas since 1984, allowing for "técnicos de cirurgía" (Assistant Medical Officers) to compensate for the severe shortages of physicians in that country. As a result, major obstetric surgeries were conducted by nonphysicians in district hospitals[12]. The "técnicos de cirurgía" program is an example of task delegation, where lower cadre of staff were substituted for higher cadre of staff. Fenton and colleagues examined potential modifiable factors that influence high maternal and perinatal mortality associated with cesarean section in Malawi. They documented the use of 45 anesthetists to conduct cesarean sections in 23 districts and two central hospitals in Malawi that helped to improve maternal health[13]. The Kenya Ministry of Health recruited and empowered committed retired or unemployed midwives living in rural areas to assist women during pregnancy, childbirth and the postpartum in their homes, manage minor complications and facilitate prompt referral when necessary to hospitals. The community midwifery program contributed to increasing the proportion of women assisted by skilled attendants in four districts of the Western Province of Kenya[14]. Thus, in sub-Saharan African countries, task delegation has had an enormous effect on increasing access to skilled attendants at birth. Following the trend towards task delegation in Ghana, community-based auxiliary health nurses (Community Health Officers or "CHOs"); have been trained as midwives to provide skilled delivery care to women in rural areas through the CHPS program.

The CHPS Program, established in 2000, aimed to improve access and quality of health care and family planning services in all the districts of Ghana[15]. The CHPS Program is implemented by the Ghana Health Service and rural communities in Ghana. The communities collaborate with the health sector in areas such as provision of land and labor for building CHPS compounds[15]. Community members also assist in providing basic health services to the people in their communities. Volunteers participate in providing health education and basic health services for minor ailments[15]. The Ghana Health Service began training a subset of basic level health care providers known as CHOs to collaborate with community members to provide skilled attendance at delivery to women in rural areas through the CHPS program[16]. A CHO is an auxiliary nurse trained for two years to provide basic health services for minor ailment such as malaria, diarrhea, headaches, family planning services, immunization and health promotion and education in rural communities and they refer complicated or advanced cases to the next level of care. A CHO-midwife is an auxiliary nurse who is further trained in midwifery for two years and by the WHO standards and certified to provide maternal health services that include skilled delivery services. The midwifery training includes basic emergency obstetric care and the basic emergency obstetric care refers to "lifesaving services for maternal complication being provided by a health facility or professional, which must include the following six signal functions: administration of parenteral antibiotics and parenteral oxytocic drugs; administration of parenteral anticonvulsants for pre-eclampsia and eclampsia; manual removal of placenta; and assisted vaginal delivery"[17].

If the CHPS Program is effective in promoting skilled attendants at delivery, it may address the human resource gap that exists for skilled delivery care in rural communities in Ghana, thereby increase the number of women who seek and receive skilled delivery services. The study assessed how and to what extent the CHO-midwifery program has been integrated into the existing CHPS. In this paper, we describe how the CHO-midwifery program has been implemented as an integrated program, and its benefits and challenges, based on the perspectives of multiple stakeholders.

## Methods

### Study setting

The study was conducted in the Kassena-Nankana East (KNE), Kassena-Nankana West (KNW), and Bongo Districts of the UER of Ghana. These districts were the first to provide skilled delivery as part of the CHPS Program. The UER population estimate from the 2010 census was 1,046,545. The KNE district had an estimated population of 109,944[18] whereas the KNW district, newly carved out of the Kassena-Nankana District in the UER, had an estimated population of 70,667 in 2012. Bongo District’s 2010 estimated census population was 84,545[18].

### Study design and methods

We employed an intrinsic case study design with a qualitative methodology. An intrinsic case study is the study of a case (e.g., person, specific group, occupation, department, organization) where the case itself is of primary interest in the exploration. The exploration is driven by a desire to know more about the uniqueness of the case rather than to build theory or how the case represents other cases[19].

We conducted in-depth interviews with community key informants such as chiefs, traditional birth attendants (TBAs), community volunteers, women leaders and elders and health professionals such as CHO-midwives, tutors of the midwifery school and the Navrongo Community Nurses School, the CHPS Coordinator, officials of the Maternal and Child Health Unit, District Directors of Health Services for the KNE, KNW, and Bongo Districts. We also reviewed annual reports of the Districts and Regional Directorate of the Ghana Health Service, UER.

### Sampling and sample size

We employed purposive sampling to select 41 stakeholders for in-depth interviews: 10 CHO-midwives, 6 CHO-midwives supervisors, 3 District Directors of Health Services, heads of maternity wards of the Navrongo Hospital, Bongo Health Centre, and Paga Health Centre, two tutors of the community health nurses and midwifery schools, two health professionals from the Regional Directorate of Health Services - UER, 15 community leaders and residents (a chief, an elder, a TBA, a community volunteer, and a woman leader from each of the three districts). We selected the program implementers based on their role in the CHPS Program and recruited the community stakeholders who were most knowledgeable about the CHPS Program.

The questions focused on the range of health services including skilled delivery services provided by CHO-midwives, how the work of skilled birth attendance is integrated with other community health services, and the successes and challenges of the new program.

### Data collection

Two research assistants were recruited from the UER and trained for the in-depth interviews. They translated the interview guides into the local languages of the three districts. They were also instructed to use tape recorders and to moderate the interviews, and they were introduced to the instructions developed for the data collection procedure. They were coached to ask questions, probe for more answers and prompt respondents for clarifications. The research assistants recorded interviews on audiotape, and a transcriber, who did not participate in interviewing, translated and transcribed the data in English. The interview formats focused on the extent to which the CHO-midwifery program has been integrated into the existing CHPS and successes and challenges of the program from their perspectives.

We pre-tested the interview guides in communities of the three districts excluded from the study, but have similar characteristics, in order to improve the relevance and appropriateness of the questions. The pre-testing was a learning session for the research assistants to improve their interviewing skills, and we revised the guides appropriately after the pre-test. Data were collected from January 13, 2012 to March 31, 2012.

### Data analysis

The analysis of narrative data on similar topics from multiple sources allows for comparison of perspectives and triangulation of reports. Members of the team (the Principal Investigator and the two Research Assistants) began the analysis by reading all interviews multiple times and discussing broad themes that emerged across respondents and areas of inquiry (integration and benefits/challenges). The team developed a coding scheme that reflected these areas and the sub-themes within each, and proceeded to code each transcript using the qualitative data software (QSR NVIVO software version 8). We produced reports on each of the broad and specific themes, which allowed us to synthesize key findings and compare responses within and between groups (e.g., community stakeholders and health professionals).

### Document review

Content analysis of program documents helped provide current and historical context in which the skilled delivery program has been implemented through the CHPS program. The investigators analyzed by objective across the documents.

### Ethics approval

We obtained ethical approval from the Navrongo Health Research Centre and the Boston University (BU) Institutional Review Boards (BU IRB reference number H-31245). We also obtained written informed consent from participants before they took part in the study.

## Results

### CHO-midwives deliver ‘integrated services’

The CHO-midwives report engagement in a wide range of services, including antenatal care, skilled delivery and postnatal care, health education, counseling, family planning services, child welfare clinics for nursing mothers, treatment of minor ailments, community durbars and other ‘outreach’ activities. They collaboratively provide these services with other nurses, community members, District Assemblies and NGO workers; community leaders confirmed a range of services the CHO-midwives and other professionals provide in their rural communities. One district chief put it this way:

….."*There is a trained midwife who assists women to deliver*, *there are also nurses*, *who take care of sick people*, *they also trained elderly women who were already delivering women to counsel pregnant women and nursing mothers and to refer pregnant women to them for deliveries*." **(Chief, KNE District)**

The CHO-midwives spoke with pride especially about the range of pregnancy and birth-related services they were now able to provide. However, if alone at their post, one CHO-midwife can provide all of the services on her own. A CHO-midwife narrates how she did it all alone.

"*I attend to outpatient department clients*, *I attend to the ANC mothers*, *I conduct deliveries and postnatal*; *when they deliver*, *I ask them to go and come back in a week*’*s time. Then I examine them to see whether the lucia is normal or not and I advise her to exclusively breastfeed the child*." **(CHO-midwife, KNW District)**

The CHO-midwives spoke thoughtfully about each aspect of care they are now able to provide.

#### Health education

According to the CHO-midwives, the health education is usually focused on a wide range of topics such as skilled delivery, antenatal and postnatal care, disease prevention, environmental cleanliness, personal hygiene, nutrition and medication. These health topics are crucial for improved health and development of mothers and children. The midwives said they usually target families, mothers, traditional leaders, TBAs, community volunteers, men and women and other stakeholders in the communities. This excerpt shows how the midwives describe their methods of health education:

….."*The community members come out for the health education and even next week we are going to have health education with the community. We have been conducting Saturday classes for the pregnant women. We tell them*, *you have to attend antenatal clinics*, *go to the clinic to deliver and go for postnatal care. We also tell them the risks involved in delivering at home*, *which includes bleeding. And if you deliver*, *we tell you to do exclusive breastfeeding*; *we educate you on how to take care of yourself so that you will not get infected. We tell you the foods that you should eat so that you will be well and healthy. We tell you how to position yourself and breastfeed the baby*." **(CHO-midwife, Bongo District)**

Also, some CHO-midwives said they provide one-on-one need-based health education and special classes to pregnant women about the importance of seeking skilled attendants at birth and the need for exclusive breastfeeding, family planning, and nutrition. They said it was important that they provided health education on individual basis to address the needs of individual women. As one CHO-midwife expressed it:

*…..*"*You know when they come for antenatal, now we don’t even do group health talks, we do individual health talks. When you come in, after examination whatever problem you find or whatever problem the woman may have, you educate her according to that. Now we no longer encourage the group discussions. It is now individual talks."***(CHO-midwife, KNE District)**

Community stakeholders also highlighted the health information and education they received from health professionals, including health talks on pregnancy, delivery, food consumption and nutrition:

*…*"*They go round to talk to us, they tell us that pregnant women should not do too much work; they also tell us the foods a pregnant woman should eat to make the mother and the baby in the womb healthy. They also encourage us to bring pregnant women to deliver in the small hospital [CHPS compound]. They tell us all these and when they go to the hospital, the nurses give the pregnant women food, children who are sent to the hospital are also given food that will make them healthy."***(Elder, KNW District)**

### Skilled birth attendance

The respondents confirmed that the CHO-midwives offer maternity services that include skilled delivery to women in rural communities. The KNE district annual report revealed that supervised deliveries increased from 63 percent in 2010 to 71 percent in 2013. And in KNW district, skilled deliveries increased from 45 percent in 2010 to 73 percent in 2013. Also, in the Bongo district, skilled attendant at birth was 96 percent in 2010 and 2013.

…. "*We offer skilled delivery services to pregnant women in this community. It is part of our job schedule and we are doing that. We are happy that we are timely intervening to prevent maternal deaths and injuries."***(CHO-midwife, KNE District)**

…. "*The maternity program here is a whole package that includes antenatal services, skilled delivery and postnatal care. When I was just a CHO, I used to provide only antenatal services and postnatal care and refer delivery cases to the health centres/hospitals. But now that I have been trained as a midwife, I supervise women to deliver and also provide other basic health services to pregnant women and other community members in my catchment area."***(CHO-midwife, Bongo District)**

Community stakeholders recognized and commended the CHO-midwives for providing skilled delivery services, but they also acknowledged that TBAs and older women provide delivery services:

…"*The midwives supervise skilled deliveries in this community. We do not need to go far to seek skilled care anymore. They are working hard to ensure that we all give birth safely and we are grateful to them. We will continue to support their work."***(Women Group’s Leader, Bongo District)**

*….."Apart from the midwife; other community members such as the TBAs and older mothers also help women to deliver in the community. These women started helping women to deliver before the midwife even came to supervise deliveries in this community."***(Community Volunteer, KNW District)**

We asked CHO-midwives about their level of confidence in providing a range of midwifery-related skills. Almost all the midwives said they had high level of confidence in providing all the services, except the manual removal of the placenta, managing prolonged labor and cord prolapse, newborn and adult resuscitation/management and use of vacuum aspirator. In the words of two CHOs:

*…*"*OK my only problem is manual removal of placenta; at least if they have to guide us to do it I will be happy, because at the facility like this, you have to know these things so that in case you are confronted you can handle the situation."***(CHO-midwife, KNE District)**

*…."For management of prolonged labor, we were taught but actually here, it is always good you refer because when the labor is long, you need to refer at this level. So even if you are trained you cannot handle it for long. We need to quickly refer because you do not have to keep someone for a long time."***(CHO-midwife, KNW District)**

Community stakeholders also admitted that midwives have limitations when it comes to provision of services in CHPS zones.

…"*We know the midwives cannot take care of every health problem that is why they sometimes refer the women to the health centers or district hospital for further investigation. For instance, a woman in my house was going to give birth and the midwife told us to send her to the hospital and when we sent her, the doctor performed an operation before removing the child. The nurse knew she could not perform the operation that is why she referred her to the hospital.*" **(Elder, KNW District)**

### Antenatal services

The KNE district 2013 annual report showed that ANC visits for first registrants increased by 1 percentage point from 77 percent in 2010 to 78 percent in 2013. The percent of women with four ANC visits increased from 49 percent in 2010 to 60 percent in 2011 and then to 64 percent in 2012 before declining to 57 percent in 2013. In KNW, the 2013 annual report revealed that ANC for first registrants was 98 percent in both 2010 and 2013. Health professionals explained that the success in attendance by first timers was due to community members’ proximity to health facilities and availability of midwives in rural communities. The CHO-midwives narrated how they encouraged pregnant women to patronize the antenatal services. They indicated that through their efforts women now visit the CHPS compounds for antenatal services:

*…*"*The successes achieved, I will talk of increases in antenatal especially first time registration because the midwives are nearby. Immediately the woman realizes that she is pregnant, she reports to the nearby clinic."***(District Director of Health Services, Bongo District)**

*…..*"*Women now come for antenatal care and I have been able to counsel people; education has improved and people’s attitudes have changed. First when I came here a woman would not come to clinic; when it was left with one month for her to deliver, then you would see her coming. Now we have educated them, when a woman misses her period she has to come to the clinic for antenatal care."***(CHO-midwife, KNE District)**

### Postpartum care

The CHO-midwife system became vibrant in 2005. Interviews with health officials revealed that the CHO-midwives provide postnatal care (PNC) to women and newborns in the three districts. According to the Bongo district 2013 annual report, PNC registration was 99 percent in 2010 and 2013. The KNW 2013 annual report indicated that PNC registration was 98 percent in 2010, but decreased to 86 percent in 2013. In the KNE district, PNC registration ranged from 86 percent in 2010 to 74% in 2013. The midwives listed an array of services they provide to make mother and newborn baby alive and healthy:

*…..*"*When a woman delivers, the CHO-midwife follows-up to ensure that the mother is healthy and able to take care of her newborn. The midwife equips her with all the information about breastfeeding, reproductive health and family planning and how to adjust to life. The midwife also educates the mother about good nutrition. These are all necessary for the good health of the mother and child."***(CHPS Coordinator, Regional Directorate of Health)**

### Referrals to health facilities

All the CHO-midwives stated that they do refer pregnant women with conditions that are beyond their expertise to the health centers or district hospitals for prompt care. We found a referral system, where transportation and other logistics were available to facilitate the referral. Health professionals reported a strong collaboration and improved communication between the CHO-midwives and the health providers at the district hospitals and health centers and CHPS compounds in the periphery. One CHO-midwife stated it this way:

*…..*"*No, normally at our level, if the labor is prolonged, you are supposed to refer to the next level; you are not supposed to manage the person at your level. We have some women, the normal hours they take, zero hour to the next twelve hours, the person should deliver, but after twelve hours if you see that the labor is not progressing you have to refer to the next level. Since I came I have referred only four labor cases and those ones, two were prolonged labor, one was underweight, below one hundred and fifty (150) and the other one was hypertensive because she had general oedema and the blood pressure was very high. I had to refer those cases because they were not my cases.*" **(CHO-midwife, KNE District)**

The midwives reported a referral system in place, but they also said the reality was that there were few, if any, ambulances to convey pregnant women from remote villages to the health centers or district hospitals. They stated that the insufficiency of transport is a major barrier to skilled birth attendance in these villages.

## Factors contributing to effective integration

### Incentives for the CHO-midwives

Interviews with health professionals revealed that the CHO-midwives are given money, study and paid leave as incentives to motivate them to continue to stay and work in rural communities. A health professional offered this view about incentives for the CHO-midwives:

*......* "*We are trying to motivate them by giving them 10% of the funds they generate from every delivery because they are working under trying conditions. The incentives are necessary to motivate them to continue to stay and work in remote areas. As I said early, it is also important to provide them with incentives because they work under harsh conditions"***(CHPS Coordinator, Regional Directorate of Health)**

A supervisor suggested that the midwives should be given incentives, awards, transfers, and logistics to work as a motivation for them to continue to reside and provide the services in rural communities.

……. "*They should motivate the midwives by giving them incentives and awards; those who perform well should be given awards. I also think they should be changing them from time to time, may be if they work in the villages for some time, one or two years, they should be sent to town and others will also go to the rural communities. They should motivate them; the managers do not see, they do not motivate people; they do not come to visit you from time to time to see your problems. Even if you have problems, the other time we had a meeting and they talked of how some of them do not even have disinfectant, something like bleach and gloves, that people were referring patients to health centers and hospitals because they do not have gloves. I am trying my best [referring to the CHO-midwife] to work and you would not supply me with the logistics to work with. So the motivation is even in the things that you work with; if you are working and all the things you need are there, you are motivated. You are in a good position to work well, but if you are working and some logistics are not there to work with, you cannot work effectively. Sometimes you are compelled to use your money to buy some of the logistics because you want to save life".***(CHPS Coordinator, Regional Directorate of Health)**

One midwife-in-charge noted another kind of incentive for CHO-midwives—the provision of compound furnishings and social amenities to enable them stay and work in rural areas in comfort:

*…..*"*You know in some places like down south they furnish the CHPS compounds for the midwife: there is a TV, a fridge, even this Multi TV. At least if you are comfortable enough, certain times if it is not all that far where your children can go to school, you can conveniently take them along to the CHPS compound. But our situation here is different because we are in remote and deprived areas, so we have to be motivated to stay here and work."* (**Midwife In-Charge of Paga Health Centre, KNW District)**

Communities have observed the efforts these midwives are making to bring health services to their doorsteps. Many believe that if the CHO-midwives are provided with the necessary tools and comfort, they will stay and deliver better services in the CHPS zones. A traditional leader also emphasized the need to motivate the midwives for optimal performance, and suggested motorbikes as an incentive that would also help them do their work more efficiently:

*……*"*If the nurses are working and nobody seems to see what they are doing they will not be happy; so I think they should be motivated to stay and work. There should be means of transport such as motorbikes. There is only one motorbike for the nurses; this one has to go there and another one wants to go somewhere else. If there are two motorbikes it will help them to work better and that will be a motivation to them."***(Chief, Bongo District)**

### Upgrading of the CHO-midwives certificates to diplomas

The CHO-midwives and other health professionals proposed that a CHO-midwife, who has a certificate in midwifery with many years of work, should be given top-up courses to receive the diploma in midwifery. This will improve their skills and serve as an incentive for many more CHOs to enter the profession. They argued that some of the CHOs prefer the mental health or the health promotion programs to the midwifery program for the reason that they will obtain a diploma after they complete the former. A CHO-midwife highlighted these points:

*…*"*Most of the community health nurses know that if they go for midwifery, they will be given a certificate so they prefer going to do mental health or health promotion to get a diploma." They could give CHO-midwives with long service top-ups courses to receive the diploma in midwifery."***(CHO-midwife, KNE District)**

### Human resources

Health professionals in our study reinforced the well-known fact that midwives and other category of nurses are in short supply in rural communities. They mentioned ageing midwives, who will soon be going on retirement, as one of the human resources problems confronted by the Ghana Health Service. One CHO-midwife expressed the dilemma in this way:

..*.*"*I think more midwives should be deployed into the communities; we have Community Health Compounds without midwives even though others may like to actually deliver in the facility, there is no midwife and the places are far. When we take Gia, Pindaa, for instance, a woman being in labor, she will actually like to go to a health facility for delivery, but you can imagine the distance. Then transportation, if Pindaa had a vehicle, a woman in labor could be transported to a health facility, but in this case, you will not get it; may be a motorbike, a bicycle which will not be so good for a laboring woman. So if midwives are around, I think we can get most of our pregnant women to come and deliver in the CHPS compound."***(CHO-midwife, KNE District)**

*….."The nurses are few; these days they use the youth employment nurses and they are not as qualified as the real nurses. They should train more nurses for the skilled delivery program."***(Community Volunteer, KNW District)**

CHO-midwives emphasized the value of the team approach to the integrated service model, as exemplified in the words of one interviewee:

*……."You know, we work together, we work as a team. I am not alone, I have CHOs with me; so if I am here running antenatal or consulting, they will be doing home visiting or the school health or even the outreaches, they run. So I do not do it alone, we work hand in hand. We work as a team."***(CHO-midwife, KNE District)**

Although most community members mentioned that the nurses were friendly, sympathetic, nice, caring and respectful towards their patients/clients, other community stakeholders also complained of the bad attitude of few nurses that included yelling at patients/clients:

*……"Some women complain that some nurses shout at them and you know it is not everyone who feels comfortable with that. You may be saying something good to someone but if you say hey!! The person’s heart will jump. So whatever you say to the person, you will only be singing. So the nurses should exercise patience. You know, a pregnant woman is like a hungry person; a hungry man is an angry man. So when a woman is pregnant, it makes her angry without any reason. The nurses should exercise patience when they are explaining things to them."***(Community Volunteer, KNW District)**

Despite the variation in the perceived quality of the CHO-midwives caring qualities, there was widespread consensus among all stakeholders that more are needed, and that the method of recruitment and training must not only ensure that their care is reliable and safe, but that they are kind and dedicated to the communities in which they live and work.

### Infrastructure strengthening

According to the midwives and their supervisors, the CHPS compounds were initially built to offer basic services excluding delivery services. Now that the compounds are used for maternity care, CHO-midwives stated that building of delivery and resting rooms are vital for effective and efficient service delivery.

…."*The only challenge I have is the delivery room: that small room here is used as consulting room, counseling and everything and also for conducting deliveries is not a convenient place. That is my challenge because I cannot put up my best because of how the room is congested with things. I am always afraid of patient or me contracting infection. I seriously need a delivery room to enable me provide efficient services*."**(CHO-midwife, KNW District)**

……*"I will say when a woman finishes delivering; we do not have a place to keep this woman. It is a limitation; she should be here for at least twenty-four hours which we cannot do now because we do not have resting room."***(CHO-midwife, KNE District)**

### Recommendations for improvement

In all of our interviews with community members and health professionals, we asked them to offer ideas for the improvement of the CHPS integrated program. Their responses fell into four main categories: 1) increase the number and quality of trained CHO-midwives, including offering refresher trainings; 2) upgrade their credential from certificate to diploma; and 3) expand infrastructure and logistics for efficient and effective service delivery; 4) and improve accommodation and social amenities available to the CHO-midwives ‘on the job’.

One CHO-midwife said creating a friendly environment, interacting with clients, refresher trainings for midwives will significantly improve the program in rural areas:

*….*"*CHO-midwives should always be at post, be friendly with our pregnant women and take good care of them [patients/clients]. If you are friendly with them, they will trust you and will always come to you. A CHO-midwife should target her pregnant women and visit them once in a while. They should give the CHO-midwives refresher trainings because there are always new ideas. The CHO-midwives on their part should learn Information Communication Technology and go to the internet for information on their work. They should buy computers for all the facilities. They should give CHO-midwives the opportunity to go for further training."***(CHO-midwife, KNW District)**

### Lessons learned

We asked the CHO-midwives and other health professionals to summarize the lessons they had learned from the initial roll out of the CHO-midwives in the CHPS zones. We discovered one overarching theme—the importance of being residents in communities. Residing in communities, CHO-midwives are able to interact with the people and know their problems, improve their relationship with community members, and thereby offer better health service. Interestingly, the CHO-midwives reported that these stronger relationships and ‘inside knowledge’ also sharpened their skills and confidence as health practitioners. The following comments reflect this theme:

*….*"*It is good, we have experienced a lot. At first you know when you are in the health center, the people come to you; you go to them on rare occasions, but now we are in the community, we do our work, we interact with them, they come to us at any time, we go to them at any time and in fact now the work is so good that I do not even know what to say."* (**CHO-midwife, Bongo District**)

*….*"*I have learned a lot because we are always with the women; and if you know your women, you will know their problems and you will help them solve the problems. Instead of coming once a while, doing your antenatal and going away, you are with them in the community and they know you, wherever they meet you they are always happy. So you interact more with your women."***(CHO-midwife, KNW District)**

*…" I work alone here and every day I attend to pregnant women and nursing mothers. The work I do daily as a midwife has made me more skillful and confident. The women also have confidence in me attending to their maternal health needs."***(CHO-midwife, KNE District)**

## Discussion

The Ghana Health Service has been challenged by a dearth of adequately trained skilled birth attendants. Our results indicate that it is possible to integrate skilled birth attendance into the role and practice of existing CHOs in rural communities in Ghana, and that their integration into existing community-based health services is viewed as making an important contribution to increase skilled birth attendance and reduce maternal death. Our findings revealed that the midwives provided services that included health education, antenatal care, skilled delivery, postnatal care, treatment of minor ailments, and participated in community durbars and other ‘outreach’ activities. The CHO-midwives often provided the services alone in situations where they were the only health professionals in the community or with other CHOs if they were in a group. This gave the midwives the opportunity to meet a wide range of people and address a wide variety of health problems, as well as to interact with diverse community stakeholders and talk about skilled care.

We believe the ‘integrated service’ strategy employed in Ghana is unique among task delegation initiatives aimed at increasing skilled birth attendance in rural communities with high rates of maternal mortality. The program gives the midwives the chance to provide varied services that include maternity care, while actively involving communities in the provision and utilization of the services. The training of CHOs as midwives and their placement in rural Ghana is timely. Our results suggest that the strategy may be a way to improve access and use of maternal health services in rural areas throughout Ghana.

In Ghana, the main objective of the skilled delivery program is to reduce maternal mortality and morbidity[20]. Historical evidence and social research have pointed to an association between skilled delivery care and maternal death and disability[4, 21–23]. In our discussions with health professionals and community stakeholders, many reported that CHO-midwives have helped prevent maternal mortality in their communities by timely intervening in maternal cases that would have resulted in fatalities. A systematic review also indicated that community-level interventions of improved perinatal care practices could bring about a reduction in maternal mortality[24]. As midwives in rural communities, they provide basic health services that include maternal health services to women in remote communities. They also promptly refer pregnant women with complications to the next level of care. Our findings suggest that antenatal attendance, skilled delivery and postpartum care were very high in the region and that could be attributed to the availability and access to maternal health services in these settings. These findings corroborate other studies from rural areas of the UER which the level of skilled delivery is higher than in other rural areas in Ghana[6, 25].

Our conversations with multiple stakeholders point to the pivotal role played by the CHO-midwives in the successful integration of basic health services and skilled birth attendance. As frontline providers in rural areas, they are sometimes called "doctors" because they are mostly the only or among the few providers of health care in rural communities. Their status as the only or among few providers and their proximity to rural communities offers them the chance to know and understand their clients/patients health problems and address them promptly. The midwives’ activities in the communities are perceived as contributing to women access and use of skilled delivery services in rural areas.

The midwives conduct normal deliveries and refer pregnant women with complications such as prolonged labor, underweight and hypertensive women, and women with previous cesarean section or multiple births to the next level for care. Our study found that CHO-midwives, by and large, refer pregnancy cases that are beyond their expertise to the health centers or district hospitals for prompt care. A strong collaboration between the CHO-midwives and the health providers at the district hospitals and health centers might have contributed to the referral of clients from CHPS zones to the next level. This facilitates easy transfer of pregnant women to the next level for care and boosts the confidence of communities in the health system to continue to send their families for care. Our results contrast with findings from rural Niger that revealed that nurses were reluctant to refer patients to hospitals for fear of loss of power and respect[26]. Our study reinforces the importance of training midwives well, and facilitating a strong collaboration between these midwives and staff of district hospitals/health centers, a strong referral system and a monitoring and evaluation system, all of which strengthen prompt referral of cases that are beyond the expertise of these midwives.

### Incentives and challenges: Maintaining a strong CHO-midwifery workforce

Our study identified strengths and limitations in the program’s sustainability. The government has put in place a set of financial and non-financial incentives to maintain a stable workforce. At the same time, underlying infrastructure gaps block the ability of the program to reach its potential in meeting the needs of rural birthing women.

### Strengths

Financial and non-financial incentives are a sure way of improving motivation and performance of health workers[27]^.^ CHO-midwives are given 10 percent of funds generated from every birth to motivate them to continue to provide the services. Our study also revealed that CHO-midwives received non-financial incentives such as "respect" and "recognition" by community members, paid leave, training, promotion and feedback from supervisors. The Government of Ghana collaborated with other partners to institute a fund for skilled delivery care to enable women access maternity services. Skilled delivery is, therefore, free for pregnant women; but the government pays the health sector for every delivery conducted. The arrangements give the CHO-midwives some financial incentive, but may sustaining the governmental support presents a challenge to an already over-burdened health care system? Perhaps, if government support ends, women in rural areas might not be able to afford the delivery fee and CHO-midwives may no longer have financial incentives to enhance their service delivery. In Kenya and other East and Southern African countries, health facilities used financial incentives to motivate their staff to continue to stay and work in the public sector[28]. In Bangladesh, removing financial barriers from users of maternal health services and providing incentives to skilled care providers helped to increase the use of skilled attendants at birth[29].

It is important that CHO-midwives are motivated as they encounter hazards in the communities and must make the sacrifice of leaving their families to work in rural settings. However, it is necessary for the Ghana Health Service, NGOs and communities to find a more sustainable way to motivate these health professionals for better service delivery

### Study limitations

The research included limited number of respondents, some selected based on the virtue of their role in the CHPS program and community. The small numbers and the uniqueness of the setting might not make the findings generalizable to other settings. On the other hand, the open-ended interview techniques allowed us to capture the views of the respondents in their own words. The triangulation of information from the in-depth interviews and document review strengthened the findings. This study is focused on skilled delivery program within the context of the CHPS and might not be generalizable to other contexts because of the uniqueness of the design and implementation of the CHPS program in the UER. However, our findings could still be adapted by other developing countries for maternal health programs. Another limitation of the research was the absence of quantitative data to determine the increase in some maternal health services, but the records review still gives a picture of trends in antenatal attendance, skilled deliveries and postnatal care in CHPS zones in UER.

### Challenges

Health professionals and community stakeholders mentioned challenges facing the program. Above all, there are many aging midwives in the system, who would be going on retirement soon. In addition, there are inadequate numbers of midwives in the health care system as well as other personnel shortages. All of these limitations lead to over-burdening the CHO-midwives in the field. The training of midwives for the program is slow coupled with attrition of some of the already trained midwives to other countries in search for jobs with better salary conditions[8]. Consequently, this phenomenon has created a wide gap between the demand for midwives in rural communities and the supply of trained midwives in the country.

Poor accommodation for midwives and other staff in the CHPS centers, lack of delivery and resting rooms for pregnant women and nursing mothers, lack of toilet facilities, bathrooms, electricity, water, vacuum aspirators and long gloves in some CHPS compounds are hindering the provision of skilled delivery services in rural communities. The Ghana Health Service started the CHPS program with the aim of offering basic healthcare to rural families, but if the midwifery program is added to CHPS, that would involve expanding the infrastructure to cater for these services. Our inquiries revealed that there were few, if any, ambulances to convey pregnant women from remote villages to the health centers or district hospitals. The health officials, CHO-midwives and community members stated that the insufficiency of transport was a major barrier to skilled birth attendance in these villages. That weakens the referral system and puts pregnant women and nursing mothers at risk of death or disability and that might reduce the confidence communities have on the health system. A study in Ethiopia also found that many women died while waiting for transportation or in the process of being transported to first referral level facilities because of the inadequacy of emergency transportation[30].

Nearly all the CHO-midwives reported that they were not well trained in the management of cord prolapse and the majority said they could not provide newborn and adult resuscitation/management and management of prolonged labor. This could threaten the provision of efficient health services in rural communities if steps are not taken to strengthen the midwifery training program.

The findings also indicate that the attitude of some nurses towards their clients/patients is appalling, which prevent some pregnant women from seeking skilled delivery services. The reason may be the pressure on these midwives to provide a wide range of services all alone. Also, the inability of the Ghana Health Service and communities to offer them enough motivation to sustain their momentum to continue to provide the services to the best of their ability could also be a major contributory factor for their behavior. Mills also reported the attitude of nurses as a major barrier to women accessing skilled delivery services. According to the report, midwives became offended if pregnant women presented late or appeared dirty or when they delayed in pushing during the second stage of labor (Mills S: *Utilization of Obstetric Services in Northern Ghana: A Quantitative and Qualitative Assessment of Skilled Health Professionals at Delivery, unpublished PhD thesis* Johns Hopkins University School of Public Health).

## Conclusions

The CHO-midwives are in CHPS compounds providing integrated health services including midwifery in rural areas. They have successfully collaborated with community members, District Assemblies and NGOs to bring health services to the doorsteps of the people and the program has achieved remarkable results in skilled care at birth. Our study demonstrated that CHO-midwives could be deployed to rural areas to provide skilled delivery services at the doorsteps of rural households. The integration of the skilled delivery program with the CHPS program appeared to be an effective model for improving access to skilled birth attendance in rural communities of the UER, Ghana.

The primary challenges that remain include inadequate numbers of CHO-midwives, insufficient transportation, infrastructure weaknesses, lack of some essential midwifery skills, abysmal attitude of the midwives, and lack of sustainable sources of incentives for health professionals. Each of these challenges requires government attention and resources.

While the Ghana Health Service should address the challenges above for program effectiveness and efficiency, further research is still needed to understand the extent to which the CHO-midwifery program has helped reduce maternal mortality and assess the cost-effectiveness of the program to guide the scale-up of the program to the rest of the CHPS zones in Ghana.

Once these barriers are addressed, deploying CHO-midwives as part of the CHPS program can be scaled up throughout all rural areas in Ghana as a key strategy to improve access to skilled attendance at birth and reduce maternal deaths throughout the nation.

### Further key recommendations

These key recommendations could further enhance the effectiveness of the midwifery program in CHPS zones:

-  The midwifery schools should train the midwife trainees in manual removal of the placenta, adult resuscitation/management, and management of cord prolapse, prolonged labor and use of vacuum aspirator to provide efficient services, especially in emergency situations.
-  The Ghana Health Service should provide the CHO-midwives with extended pre-and in-service training and the needed logistics to improve the range and quality of services they provide in the CHPS zones. They should organize periodic in-service training for the midwives to equip them and update them with new ideas and skills. The Ghana Health Service should offer the CHO-midwives with information, communication and technology literacy training to be abreast with world issues and to learn new things about their career.
-  The CHO-midwives should continue to intensify health education about the need for skilled delivery care in rural areas. Families should be targeted with health education to promptly refer or accompany pregnant women for maternal health services.
-  In order to provide space for delivery and enable the midwives detain and monitor newly born children and their mothers for 24 hours before they are discharged, the Ghana Health Service should consider including delivery and resting rooms when they build new CHPS compounds or expand old ones.
-  The Ghana Health System, the regional medical stores should ensure regular supply of drugs and other logistics to the CHPS compounds to guarantee the continuation of the program and build community confidence on the operations of the system.
-  The Ghana Health Service should assign ambulances to the CHPS Program for prompt transportation of women with complications to the health centers and district hospitals. They could collaborate with the government of Ghana, NGOs, Civil Society and individuals to purchase these ambulances for the program.
-  The Ghana Health Service, the District Assemblies and NGOs should provide adequate accommodation, bathrooms, toilet facilities, water, electricity and television for the health professionals to offer them comfort and motivate them to continue to reside and work in rural areas.
-  The Ghana Health Service, NGOs, communities and individuals should provide financial incentives to CHO-midwives to motivate them to stay and work in rural communities. The Service is already giving the midwives 10 percent of funds generated from every delivery and communities and other stakeholders should contribute to this fund, but the Service should identify a more sustainable way of rewarding these midwives. However, the Ghana Health Service should carefully design, manage, monitor and evaluate these incentives to measure their impact on the midwives’ work performance.
-  Apart from the paid and study leave and the monetary incentives, the Ghana Health Service should award and appreciate CHO-midwives in other forms such as offering them a certificate of recognition for best performance.
-  Communities should also motivate the CHO-midwives to continue to stay and work with them. They could assist the midwives in their house chores and farms. This will help strengthen the partnership between the midwives and community residents for better service delivery and utilization.
-  The Ministry of Health and the Ghana Health Service should clearly define the career path of this cadre of nurses. The CHO-midwives, who have the midwifery certificate with long duration of service, should be given top-up training to receive the diploma in midwifery, but this should be done carefully to ensure that standards are met.
